# Clinical Manifestations and Genomic Evaluation of Melioidosis Outbreak among Children after Sporting Event, Australia

**DOI:** 10.3201/eid2911.230951

**Published:** 2023-11

**Authors:** Simon Smith, Tonia Marquardt, Amy V. Jennison, Andrew D’Addona, James Stewart, Trent Yarwood, Jennifer Ho, Enzo Binotto, Julian Harris, Mark Fahmy, Juliet Esmonde, Megan Richardson, Rikki M.A. Graham, Richard Gair, Lawrence Ariotti, Annie Preston-Thomas, Sally Rubenach, Siobhan O’Sullivan, Darren Allen, Thomas Ragh, Sachjuan Grayson, Sophie Manoy, Jeffery M. Warner, Ella M. Meumann, Jennifer M. Robson, Josh Hanson

**Affiliations:** Cairns Hospital, Cairns, Queensland, Australia (S. Smith, J. Stewart, T. Yarwood, J. Ho, E. Binotto, J. Harris, M. Fahmy, S. O’Sullivan, T. Ragh, S. Grayson, S. Manoy, J. Hanson);; Cairns & Hinterland Health Service, Cairns (T. Marquardt, A. D’Addona, J. Esmonde, M. Richardson, R. Gair, A. Preston-Thomas, S. Rubenach);; Forensic and Scientific Services, Brisbane, Queensland, Australia (A.V. Jennison, R.M.A. Graham, L. Ariotti);; University of Queensland, Herston, Queensland, Australia (T. Yarwood); James Cook University, Cairns (T. Yarwood, J. Ho);; Royal Brisbane and Women’s Hospital, Brisbane, Queensland, Australia (D. Allen);; James Cook University, Townsville, Queensland, Australia (J.M. Warner);; Sullivan Nicolaides Pathology, Bowen Hills, Queensland, Australia (E.M. Meumann, J.M. Robson);; Menzies School of Health Research, Darwin, Northern Territory, Australia (E.M. Meumann);; University of New South Wales, Sydney, Australia (J. Hanson)

**Keywords:** Burkholderia pseudomallei, melioidosis, bacteria, tropical medicine, pediatrics, public health, Australia

## Abstract

Melioidosis, caused by the environmental gram-negative bacterium *Burkholderia pseudomallei*, usually develops in adults with predisposing conditions and in Australia more commonly occurs during the monsoonal wet season. We report an outbreak of 7 cases of melioidosis in immunocompetent children in Australia. All the children had participated in a single-day sporting event during the dry season in a tropical region of Australia, and all had limited cutaneous disease. All case-patients had an adverse reaction to oral trimethoprim/sulfamethoxazole treatment, necessitating its discontinuation. We describe the clinical features, environmental sampling, genomic epidemiologic investigation, and public health response to the outbreak. Management of this outbreak shows the potential benefits of making melioidosis a notifiable disease. The approach used could also be used as a framework for similar outbreaks in the future.

Melioidosis, caused by the environmental gram-negative bacterium *Burkholderia pseudomallei*, is endemic in northern Australia (*1*). The most common clinical manifestation of the infection is pneumonia, with or without bacteremia, but almost any organ can be involved, including the liver, spleen, prostate, skin, bones, joints, and central nervous system (*2*). *B. pseudomallei* is an opportunistic pathogen that usually affects adults, ≈90% of whom have underlying conditions that predispose them to developing the disease (*1*,*3*).

Melioidosis is uncommon in children. In a large prospective series from northern Australia, children represented only 4% of cases (*1*). The infection is usually subclinical in children, and a case series from Thailand estimated that only 1 in 4,600 antibody-producing exposures resulted in symptomatic disease (*4*). When clinical disease occurs, children with melioidosis usually have limited cutaneous disease; however, invasive disease, including bacteremia and meningoencephalitis, also can occur, particularly in children with underlying conditions (*5*–*8*).

*B. pseudomallei* is saprophytic and is in soil of endemic tropical and subtropical areas. During the dry season, the organism is found at soil depths of >30 cm, but during the wet season, monsoonal rains cause the rising water table to bring bacteria to the surface where they proliferate (*9*), increasing the risk for human exposure to the organism. That bacterial cycle also explains the strong seasonality of the disease (*10*–*12*). 

Melioidosis is usually acquired through percutaneous inoculation, inhalation of contaminated dust, or ingestion of contaminated water (*1*,*13*). Although inoculation events are often not evident, many infected persons report recent recreational activities, such as gardening, or occupational exposure to soil or surface water (*1*). In Far North Queensland, a tropical area of Australia, the incidence of melioidosis in the region’s main city has increased 10-fold in the past 22 years (*14*). The reasons for the increase are not completely understood but could be related to the local construction of major infrastructure and the expansion of the urban–rural fringe (*14*).

Cases of melioidosis acquired through sporting activities are exceptionally rare, even in endemic areas where soil sampling confirms the presence of *B. pseudomallei* on sports fields, likely because sports participants lack predisposing conditions for melioidosis (*15*). Although sporadic melioidosis cases have been linked to sporting events, no outbreaks have been genomically linked to the site of the sporting event. We report the clinical characteristics, case management, and patient outcomes, as well as genomic evaluation and the public health response, for a melioidosis outbreak among children after a sporting event in a tropical region of Australia.

## Methods

### Sporting Event

On 1 day in November 2022, children in a primary school in Queensland participated in a sporting event. The event occurred at the end of the region’s dry season, when cases of melioidosis are uncommon, and minimal rain had fallen in the preceding months (*16*). The sporting event involved an obstacle course on the school grounds that included crawling through a mud pit.

Nineteen days after the event, an 8-year-old female child (case 1) was seen by her general practitioner for a 2-cm pustular lesion on her left arm. She received oral cefalexin for 5 days, but the lesion persisted, and further lesions appeared on her left leg, right leg, and back. *B. pseudomallei* was isolated from a swab of one of the lesions. 

### Public Health Response

Melioidosis is a notifiable disease in Queensland, which expedited the public health response. Pathology providers directly alerted the local public health team after culture confirmation, enabling the prompt alert of the school, the children’s parents, and healthcare providers. Those notifications encouraged parents of affected children to seek healthcare, particularly for nonhealing skin lesions. 

**Figure 1:**
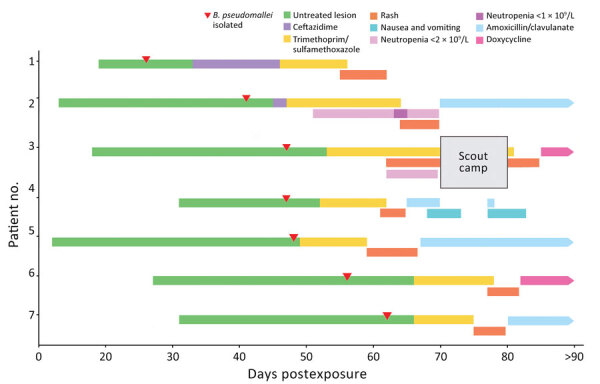
Timeline of clinical manifestations and treatment of melioidosis among 7 children after sporting event, Australia. Limited cutaneous melioidosis developed in children after crawling through a mud pit on an obstacle course in a tropical region of Queensland. All children experienced an adverse drug reaction to trimethoprim/sulfamethoxazole, the preferred oral antimicrobial agent. All case-patients had good clinical outcomes, suggesting that a shorter duration of antimicrobial drugs might be appropriate for limited cutaneous melioidosis in some children.

**Figure 2:**
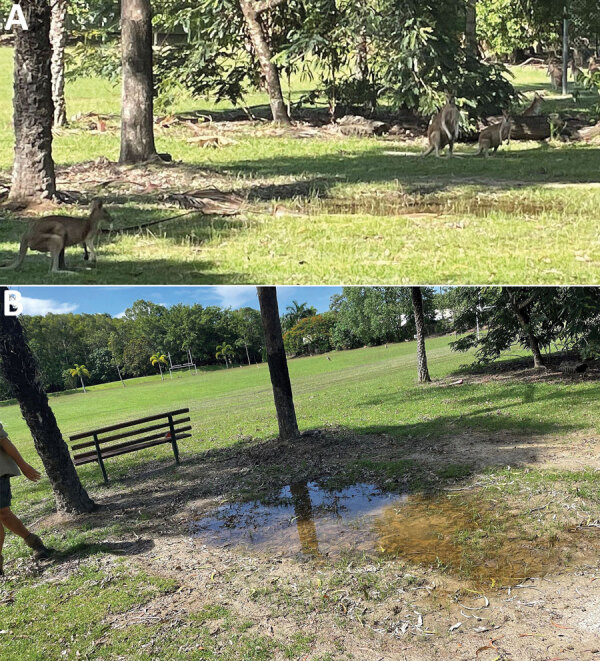
Site of exposure in an outbreak of limited cutaneous melioidosis among children after a sporting event, Australia. A) Mud pit site when not in use for the sporting event; members of a wallaby troupe surround the site. B) Mud pit site when not in use for the sporting event; water pooling is evident.

**Figure 3:**
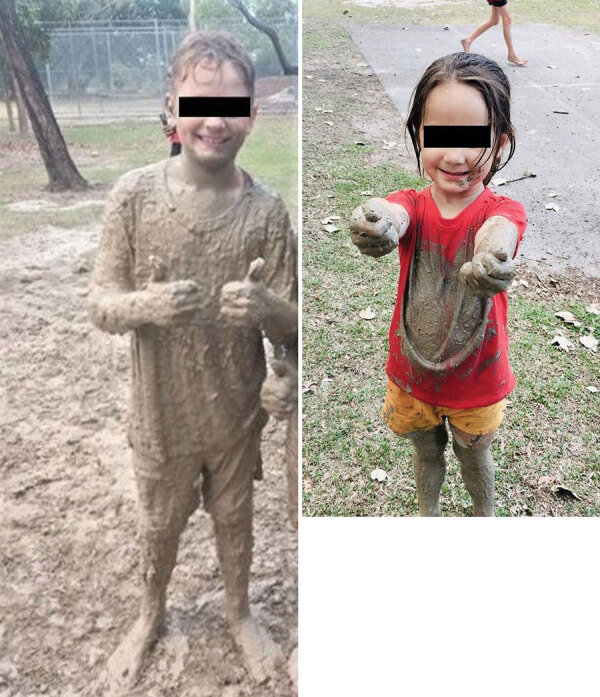
Images of participants immediately after a sporting event that resulted in an outbreak of limited cutaneous melioidosis, Australia. The sporting event was held in a tropical region of Queensland and involved crawling through a mud pit on an obstacle course. Children are extensively covered in mud immediately after participating in the event. Neither of the pictured children contracted melioidosis. However, *Burkholderia pseudomallei* was isolated in soil samples from the mud pit and genomically linked to *B. pseudomallei* isolated from cutaneous lesions on 7 children who participated in the event and had melioidosis diagnoses.

A total of 7 melioidosis cases were detected among children who participated in the obstacle course event (Figure 1). Besides the case-patients, ≈265 other students also participated in the event, which included a mud pit. The pit had been formed 10 years previously and was dug each year to a depth of ≈50 cm and filled with water. After each year’s event, the soil was returned to the pit. When not in use, the pit site had become a shallow depression that allowed water to pool (Figure 2). The rest of the obstacle course was located on undisturbed sports fields. For the November 2022 event, the pit had been filled with chlorinated tap water. All 7 case-patients had crawled through the pit multiple times during the event (Figure 3).

**Figure 4:**
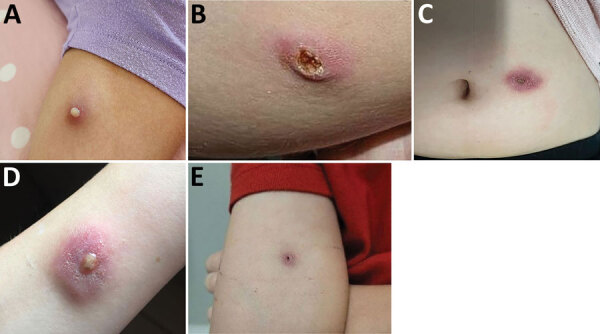
Clinical images of cutaneous melioidosis among children after sporting event, Australia. A) Cutaneous pustular lesion on the left arm of case 1; lesion initially appeared at 19 days postexposure (dpi). B) Cutaneous ulcerative lesion on the right leg of case-patient 2; lesion initially appeared at 14 dpi. C) Cutaneous lesion near the umbilicus of case-patient 3; lesion initially appeared at 18 dpi. D) Cutaneous lesion on the left arm of case-patient 4; lesion initially appeared at 31 dpi. E) Cutaneous lesion on the right arm of case-patient 5; lesion initially appeared at 12 dpi.

All 7 infected children were immunocompetent. All had limited cutaneous disease (Figure 4), and 4 were aware of a pre-existing lesion, usually insect bites, before participating in the event. None reported sustaining an injury during the event. 

Wallabies, marsupials in the Macropodidae family, had been observed on the school grounds. The wallaby troupe had increased to ≈200 members during the previous 5 years and were often seen near the site of the mud pit (Figure 2, panel A). Wallabies previously have been hypothesized to spread melioidosis through fecal shedding (*17*). None of the case-patients reported any notable interaction with the wallabies. Construction has previously been hypothesized to increase the risk of melioidosis, likely by the inhalation route (*14*), and 6 of the 7 case-patients regularly walked past roadworks taking place at the edge of the school grounds on their journey to school.

### Antimicrobial Drug Treatments

**Figure 5:**
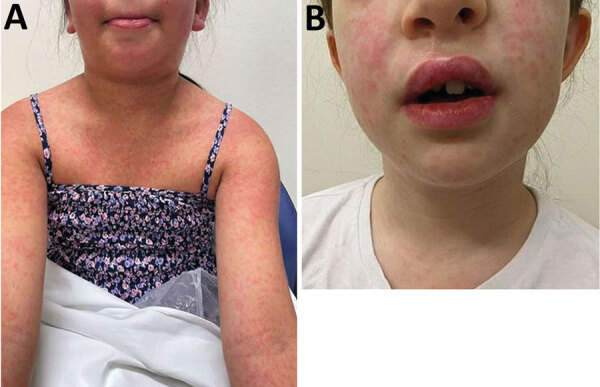
Clinical images of adverse reactions secondary to trimethoprim/sulfamethoxazole among children treated for cutaneous melioidosis after a sporting event, Australia. A) Widespread, erythematous, pruritic rash in case-patient 1 that began 9 days after commencing trimethoprim-sulfamethoxazole. B) Lip swelling and a widespread erythematous rash in case-patient 2 that began 16 days after commencing trimethoprim/sulfamethoxazole.

The clinical management of melioidosis usually consists of 2 phases: an intensive phase consisting of intravenous meropenem or ceftazidime for a minimum of 14 days, then an eradication phase of oral trimethoprim/sulfamethoxazole (TMP/SMX) for a minimum of 12 weeks (*18*). Case-patient 1 received intravenous ceftazidime for 14 days, then TMP/SMX. However, 9 days after commencing TMP/SMX, a widespread, erythematous, pruritic rash developed (Figure 5, panel A). Her antibiotics were ceased, and her rash improved after 5 days. Case 2 also had an adverse reaction to TMP/SMX (Figure 5, panel B). The remaining cases also had adverse reactions to TMP/SMX, necessitating cessation of the drug and prompting an investigation of the adverse drug event (Figure 1).

In all, 5 children received oral therapy only, and 1 received only 2 days of intravenous therapy (Appendix). Because of the adverse reactions to TMP-SMX, amoxicillin/clavulanate (AMOX/CLAV) was the predominant antibiotic used in 4 cases (Table 1). 

**Table 1:** Clinical features, antimicrobial drug treatment, and outcomes of limited cutaneous melioidosis among children after sporting event, Australia*

Case no.	Age, y/sex	Location of cutaneous lesion(s)	No. swabs†	Immunocompetent	Disseminated foci excluded‡	Intensive phase duration	Eradication phase duration§	Primary antimicrobial drug#	Outcome
1	8/F	Left arm, right leg, low back	1	Y	Y	14 d	9 d	Ceftazidime	Recovered
2	8/F	Right leg, left leg	2	Y	Y	2 d	12 wk	AMOX/CLAV	Recovered
3	10/F	Abdomen	1	Y	Y	NA	8 wk	Doxycycline	Recovered
4	7/M	Left arm	1	Y	Y	NA	17 d	AMOX/CLAV	Recovered
5	7/M	Right arm	2	Y	Y	NA	12 wk	AMOX/CLAV	Recovered
6	7/F	Right arm	1	Y	Y	NA	9 wk	Doxycycline	Recovered
7	9/F	Right hip	1	Y	Y	NA	12 wk	AMOX/CLAV	Recovered

*AMOX/CLAV, amoxicillin/clavulanate. NA, not applicable. †Number of swab samples collected before *Burkholderia pseudomallei* was cultured. ‡Defined by no *B. pseudomallei* growth in blood cultures, and no evidence of infection on chest radiograph and abdominal ultrasound. §Defined as total duration of antimicrobial drug course, excluding treatment interruptions. #Antimicrobial drug used for longest time.

### Adverse Drug Event Investigation

When a skin reaction developed in the second patient, the Therapeutic Goods Administration was notified by the hospital pharmacist involved in the care of the patient. Liquid chromatography quadrupole time-of-flight mass spectrometry of 1 patient’s urine sample detected TMP/SMX but no unexpected additional compounds. TMP/SMX tablets from separate batches, all of which had been given to the case-patients, were sent to the Therapeutic Goods Administration for further investigation, but only controlled impurities within the control limit specified in the pharmacopeia monograph were identified.

### Environmental Sampling and Analysis

**Figure 6:**
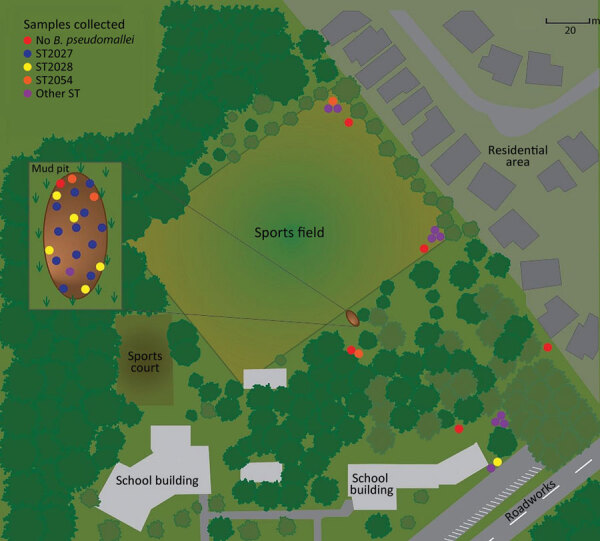
Location of sporting event that resulted in an outbreak of limited cutaneous melioidosis, Australia. The sporting event involved crawling through a mud pit on an obstacle course. Inset shows detail of the mud pit area. Colored dots indicate environmental sampling sites and *Burkholderia pseudomallei* STs detected from samples. Among participants, 7 children who crawled through the mud pit had clinical manifestations of melioidosis. *Burkholderia pseudomallei* isolated in soil samples from the mud pit were later genomically linked to isolates from cutaneous lesions on 7 children. ST, sequence type.

We hypothesized that the mud pit was the source of the outbreak and performed environmental sampling 13 weeks after the obstacle course event. Sampling occurred during the region’s wet season. We used international recommendations for sampling, but laboratory capacity limited the number of samples that we could process (*9*). In total, we obtained 18 environmental samples from various areas of the obstacle course event: 14 soil samples, 2 bore water samples from the source used to irrigate the field, and 2 separate collections of wallaby scat weighing 100 g each (Table 2; Figure 6). 

**Table 2:** Environmental sample selections and sampling methods in an investigation of melioidosis among children after sporting event, Australia

Sample site	Rationale for site selection	Sampling method	No. samples
Mud pit	Epidemiologic review suggested the mud pit used during the event was the most plausible source of infection	Soil samples were collected at a minimum of 2.5 m apart and in a grid format	8
	Because this was the likely point of acquisition, the greatest number of samples were taken here	Two additional samples taken in the center of the mud pit	
Earth works	Determine whether earth works brought into the site introduced *B. pseudomallei*	Area was condensed rock and soil, which was a barrier to reaching 30 cm depth. Soil was also well drained	1
	Identify whether runoff from this site affected other parts of the school, sports field, or mud pit area	Sample was collected at a random spot in the earth works area due to limited laboratory capacity	
	Identify whether this area had similar contamination as other areas		
Drainage area adjacent to earth works	Identify whether runoff or sediment from earthworks or school grounds contained *B. pseudomallei*	Samples were collected at random spots in sample area due to limited laboratory capacity	3
	Determine if the stormwater diversion drains were introducing *B. pseudomallei* to the school site	Sample location was identified because areas where runoff and sediment from the earthworks site and school might collect and settle	
Side sports field stormwater run off	Area appeared to hold water runoff from earthworks site and sports field	Samples were collected at random spots in sample area due to limited testing ability	2
	Detection of *B. pseudomallei* might have supported theory that *B. pseudomallei* was introduced to the school site through earthworks	Sample location was identified as an area where water runoff from earthworks site and sports field collected and pooled	
Wallaby scat	Evidence that wallabies can carry *B. pseudomallei* that might have been spread across the site through their feces (*17*)	Surface sampling of wallaby feces in areas witnessed to have a high population of wallabies grazing during sampling visit	2
		Scat collected from multiple droppings to meet the 100 g sample requirement Scat collected from ground	
Bore water pump	Evidence that bore water has been found to contain *B. pseudomallei* (*19*)	Two water samples taken, a first flush sample and then a sample after the bore had run for a 2-min period.	2
		Samples required a minimum of 1 L collected into sterile containers	

We performed culture of soil and wallaby scat, as previously described (*8*). In brief, we placed 10 g of soil or scat into 10 mL Ashdown’s selective broth (Oxoid–Thermo Fisher Scientific, https://www.thermofisher.com). For bore water, we filtered 1 L through a 0.45 μm filter (Pall Life Sciences, https://www.pall.com), then transferred to 10 mL Ashdown’s selective broth. We vigorously vortexed the broth, then incubated at 37°C for 48 hours and subcultured to Ashdown’s solid medium plates (Edwards Microbiology, https://www.edwards.com). We reviewed plates for *B. pseudomallei* morphotypes and screened up to 5 suspect colonies by using Biotyper (Bruker Corporation, https://www.bruker.com) matrix-assisted laser desorption/time-of-flight (MALDI-TOF) mass spectrometry to capture potential strain variation. 

Of the 18 environmental samples, we isolated *B. pseudomallei* from 12 (67%), which included 49 individual isolates. We screened those 49 isolates and 29 returned a result of *B. thailandensis* using the MALDI Biotyper Library (Bruker), an in vitro diagnostic (IVD) database, which cannot distinguish between *B. thailandensis* and *B. pseudomallei*. We further analyzed all IVD spectral profiles using a curated *B. pseudomallei* library and confirmed that all 29 *B. thailandensis* isolates were actually *B. pseudomallei*. The other 20 isolates returned no identification using the IVD library, but we subsequently confirmed those as *B. pseudomallei* by using the curated library. The 49 colonies displayed minimal colony variation, and we selected 32 colonies for further testing.

### Genomic Investigation

We performed whole-genome sequencing on all 7 clinical isolates and 32 environmental isolates. We extracted and prepared DNA from the isolates for sequencing, as previously described (*20*). In brief, we prepared DNA by using the Nextera XT Kit (Illumina, https://www.illumina.com) and sequenced on the NextSeq 500 using the NextSeq 500 Mid Output Version 2 Kit (Illumina) at 300 cycles, according to the manufacturer’s instructions. We used Trimmomatic version 0.36 to trim sequences (*21*), and quality checked sequences by using FastQC version 0.11.5 (Babraham Bioinformatics, https://www.bioinformatics.babraham.ac.uk) and MultiQC version 1.1 (https://multiqc.info) (*22*). We used SPAdes assembler version 3.12.0 (*23*) to perform de novo assembly of sequences into contigs. We performed multilocus sequence type (MLST) and core genome MLST (cgMLST) analysis by using Ridom SeqSphere+ version 8.4 (Ridum Bioinformatics, https://www.ridom.de) and publicly available schemes at PubMLST (*24*). We uploaded sequence data to GenBank (BioProject accession no. PRJEB61871).

**Figure 7:**
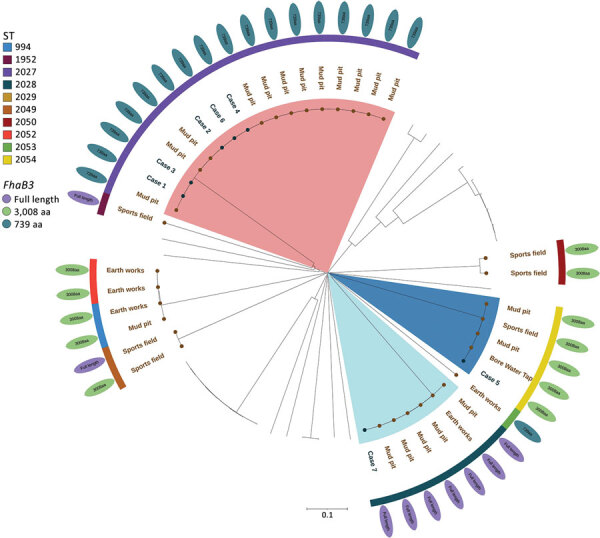
Core multilocus sequence typing of *Burkholderia pseudomallei* isolated in an outbreak of melioidosis among children after a sporting event, Australia. The phylogenetic neighbor-joining tree shows the relationships between environmental and clinical isolates; isolate sources are noted. The tree was constructed by using SeqSphere version 9.0.8 (Ridom, https://www.ridom.de) on the *fhaB3* gene and iTOL (https://itol.embl.de) was used to add the annotations. Identified *B. pseudomallei* STs are noted. Scale bar indicates nucleotide substitutions per site. ST, sequence type.

Among the clinical isolates, we found 3 different sequence types (STs): ST2027 in 5 cases, ST2028 in 1 case, and ST2054 in 1 case. Environmental isolates showed much more diversity; we found 9 different STs, 3 of which matched STs of the clinical isolates (Table 3; Figure 7). Genomic analysis of cgMLST showed isolates of all 3 STs from the mud pit and the children had 0–2 cgMLST allele difference, a level of genetic similarity consistent with the mud pit being the source of the exposure (*25*).

**Table 3:** Microbiological and genomic results from environmental testing of *Burkholderia pseudomallei* from investigation of melioidosis outbreak among children after sporting event, Australia

Sample site	No. samples collected	No. samples growing *B. pseudomallei*	No. *B. pseudomallei* isolates									
				Sequence type								
				2027*	2028*	2054*	994	2052	2053	2049	2050	1952
Mud pit	8	7	20	12	5	2	1					
Earth works, including drainage	4	2	5		1		1	2	1			
Sports field	2	2	6			1				2	2	1
Wallaby scat	2	0	0									
Bore water	2	1	1			1						

*Sequence type genetically matching a clinical case.

We used BLAST (https://blast.ncbi.nlm.nih.gov) analysis to investigate the *fhaB3* gene, a virulence factor involved in host cell attachment and associated with bacteremia, against full-length *fhaB3* genes from the *B. pseudomallei* K96243 genome (GenBank accession no. NZ_CP009537) (*26*). Of the clinical cases, we found ST2028 isolates had the full-length *fhaB3* gene, but other STs had a truncated version of *fhaB3* that reduced the peptide length from 3,103 to 739 amino acids in ST2027 and to 3,008 amino acids in ST2054 (Figure 7). We are uncertain of the potential functionality of these truncated versions but suspect they might not have the same functionality as the full-length version.

## Discussion

This outbreak of melioidosis originating from a sporting event is striking for several reasons. Although the outbreak occurred in an endemic area, the attack rate was 2.6%, much higher than the 0.02% rate of symptomatic disease reported in seropositive children in Thailand (*4*). The difference could partly be explained by the higher rates of seropositivity generally seen in children in Thailand, which potentially results from increased exposure to *B. pseudomallei* from ingestion of unchlorinated tap water in early life (*27*,*28*). In the outbreak we describe, existing abrasions and minor skin trauma sustained during the obstacle course might have enabled inoculation in the mud pit. Our environmental testing was not able to quantify *B. pseudomallei* in the mud pit samples, but a larger inoculum, which can contribute to the development of disease, might be partly responsible for the increased number of cases (*29*). The full and truncated *fhaB3* gene or other undefined virulence factors also might have contributed to the higher attack rate, although absence the *fhaB3* gene has previously been correlated with cutaneous disease (*26*). In addition, limited cutaneous melioidosis in immunocompetent children occasionally will heal spontaneously (*6*). Public health messaging and greater awareness in this outbreak might have improved the detection of cases in persons who otherwise would not have sought medical attention.

The exposure event occurred at the end of the region’s dry season, when cases of melioidosis are uncommon, highlighting that human modification of the environment can increase exposure to *B. pseudomallei* (*30*,*31*). MLST matching of the children’s *B. pseudomallei* isolates to those found in the mud pit confirmed that the pit was the source of the outbreak. Contaminated unchlorinated bore water supplies have been implicated in other melioidosis outbreaks (*19*). In this outbreak, bore water was used regularly on the school’s sports fields, but it had not been used to fill the mud pit. However, sampling of the bore water did identify an MLST that matched 1 child and 2 samples from the pit, suggesting that both the pit and the bore might have become contaminated by organisms from the surrounding soil. For this sporting event, the pit had been filled with chlorinated tap water, but we were unable to ascertain if a different water source was used in previous years. In addition, because of resource limitations, we were unable to collect extensive environmental samples, including from irrigation equipment that might have been used to fill the pit.

In 2 of the children, an initial swab sample tested negative. Culture of *B. pseudomallei* remains the standard for diagnosing melioidosis, and those cases highlight that even in well-equipped laboratories, multiple or repeat swab samples from skin lesions might be needed to confirm the diagnosis (*32*). When melioidosis is clinically suspected or a history of soil or water exposure exists, clinicians should advise the laboratory so that appropriate selective and differential media can be used.

Although the same pit had been used in the obstacle course for 9 years before this outbreak, participation in the event had not previously been associated to any confirmed melioidosis cases. However, repeated use and subsequent ground sinkage and water pooling could have resulted in greater soil water content, creating an optimal environment for *B. pseudomallei* growth (*33*). No *B. pseudomallei* was detected in the 2 wallaby scat samples, making the presence of a large troupe of wallabies an unlikely explanation for the outbreak. However, testing of further samples would be required to fully exclude wallabies as the source of *B. pseudomallei* (*17*).

The clinical management of melioidosis usually consists of an intensive phase of intravenous meropenem or ceftazidime for a minimum of 14 days, then an eradication phase of oral TMP/SMX for a minimum of 12 weeks (*18*). However, an eradication phase–only regimen using oral TMP/SMX has been proposed for limited cutaneous disease in children without risk factors for invasive disease and without disseminated foci (*6*). In our series, 5 children received oral therapy only, and 1 received only 2 days of intravenous therapy. Also, because of the adverse reactions to TMP/SMX, AMOX/CLAV was the predominant antibiotic used in 4 cases. In adults, AMOX/CLAV is associated with higher relapse rates than TMP/SMX–based regimens (*34*), but AMOX/CLAV has been successfully used as eradication therapy in some children (*28*). However, the recommended dosing of 20 mg/kg amoxicillin and 5 mg/kg clavulanate 3 times a day could influence tolerability and adherence (*35*). In 2 cases, doxycycline was the predominant antimicrobial drug therapy, and both children recovered. Doxycycline has previously been avoided in children <8 years of age because of concerns about dental staining, but durations <21 days are considered safe in any age group. Rarely, longer courses of doxycycline can be required in children when no suitable alternative is available (*36*). However, adverse events, such as erosive esophagitis and photosensitivity, particularly in tropical climates, require consideration. In our cases, a doxycycline dose of 2 mg/kg twice daily was administered and was well tolerated by the children.

The clinical course of the children in this outbreak supports recommendations that oral antibiotics alone are appropriate for children with limited cutaneous melioidosis (*6*). Although TMP/SMX remains first-line therapy, alternative agents can be substituted if TMP/SMX is not tolerated. A 3-month course is recommended for limited cutaneous disease, but recovery is possible with shorter durations (*6*,*28*). In this outbreak, 4 cases received substantially less than 3 months of oral therapy, suggesting that, in immunocompetent children without signs of dissemination, a shorter duration could be sufficient (*37*).

Melioidosis is extant throughout tropical regions of the world, and outbreaks could occur in areas not yet considered endemic for the disease. However, acquiring melioidosis from participation in sporting activities remains exceptionally uncommon, and the risk to children playing in mud or surface water in melioidosis endemic areas is infinitesimally small. Furthermore, that none of the 7 cases in this outbreak developed into invasive disease and that all case-patients had a good clinical outcome is reassuring. Nonetheless, these cases highlight some of the challenges in the diagnosis and management of melioidosis, and the adverse reactions to TMP/SMX illustrate the potential risks associated with this antimicrobial agent (*18*). These cases also suggest that a shorter duration of antibiotics for limited cutaneous melioidosis might be appropriate for some children, and the use of doxycycline, a drug often avoided in children, could be useful in children with melioidosis if TMP/SMX is not tolerated. As a result of this outbreak, Far North Queensland guidelines have been updated to include advice about the risk to participants of events that include exposure to deeper layers of soil, and consideration of risk assessment for such activities. 

In conclusion, the management of this outbreak highlights the virtue of making melioidosis a notifiable disease. The approach used for the public health response, environmental sampling, and genomic investigation of a melioidosis outbreak provided here could be used as a framework for similar outbreaks in the future.

Appendix Additional information on clinical manifestations and genomic evaluation of melioidosis outbreak among children after sporting event, Australia.
