# Supplementary material for: Clinical Manifestations and Genomic Evaluation of Melioidosis Outbreak among Children after Sporting Event, Australia
Source: Emerg Infect Dis. 2023 Nov;29(11):2218–28. doi: 10.3201/eid2911.230951 (PMC10617349; doi:10.3201/eid2911.230951)
Supplement: Appendix — Additional information on clinical manifestations and genomic evaluation of melioidosis outbreak among children after sporting event, Australia. [file 23-0951-Techapp-s1.pdf]

Article DOI: https://doi.org/10.3201/eid2911.230951

EID cannot ensure accessibility for supplementary materials supplied by authors. Readers who have difficulty accessing supplementary content should contact the authors for assistance.

Clinical Manifestations and Genomic Evaluation of Melioidosis Outbreak among Children after Sporting Event, Australia

Appendix

Detailed Clinical Course of Each Case

Patient 1. An 8-year-old female attended her general practitioner (GP) with a 2 cm pustular lesion on her left arm. She received oral cefalexin for 5 days, but the lesion persisted, and further lesions appeared on her left leg, right leg, and back. A swab of one of the lesions isolated *Burkholderia pseudomallei*. She received intravenous ceftazidime for 14 days followed by oral trimethoprim-sulfamethoxazole (TMP-SMX). However, 9 days after commencing TMP-SMX, she developed a widespread, erythematous, pruritic rash. Her antibiotics were ceased, and her rash improved after 5 days. At this point, the original pustular lesions were resolving, and a decision was made to avoid further antibiotics. At 4 months all the lesions had resolved entirely.

Patient 2. An 8-year-old female attended her GP with a painful, purulent 1.5 cm ulcer on her right leg and a second, smaller left leg lesion. She was prescribed topical mupirocin followed by oral cefalexin without improvement. An initial swab identified no organisms, but her GP was aware of patient 1 and collected a second swab 8 days later and *B. pseudomallei* was isolated. She received intravenous ceftazidime which was ceased after 48 hours as blood cultures were negative. She was prescribed oral TMP-SMX but developed neutropenia (1.85×10^9/L; reference interval $2.0–8.0 \times 10^9$/L) on day 6. TMP-SMX was continued but on day 16, she developed lip swelling, a widespread erythematous rash, and neutropenia to 0.68×10^9/L; TMP-SMX was ceased. Her rash resolved after 6 days, and she was commenced on oral amoxicillin-clavulanate. She completed 12 weeks of antibiotics and her lesions resolved entirely.

Patient 3. A 10-year-old female attended her GP with a 2 cm purulent lesion near her umbilicus. She was prescribed oral cefalexin with minimal improvement; a subsequent swab isolated *B. pseudomallei* and *Staphylococcus aureus*. She was prescribed oral TMP-SMX but after 10 days developed an erythematous rash on sun-exposed areas and neutropenia (1.6×10^9/L). Her TMP-SMX was continued but ceased 1 week later when she attended a scout camp. On her return, she recommenced TMP-SMX but within 30 minutes developed generalized pruritis, vomiting, and a widespread erythematous rash. She attended hospital where she was febrile and a TMP-SMX drug reaction was suspected. Her TMP-SMX was ceased and her rash resolved after 4 days. Due to family concerns about the size of amoxicillin-clavulanate tablets, she was prescribed oral doxycycline. She developed photosensitivity but otherwise tolerated doxycycline well, her lesion resolved, and she completed a further 7 weeks of therapy.

Patient 4. A 7-year-old male attended his local doctor with a 3-week history of a 2 cm abscess on his left forearm and a febrile episode 2 weeks previously. The abscess was lanced and a swab isolated *B. pseudomallei*. He was prescribed oral TMP-SMX but after 9 days developed a diffuse, erythematous rash. His TMP-SMX was ceased, and his rash resolved after 3 days. He was commenced on oral amoxicillin-clavulanate but developed diarrhea and anorexia and ceased this after 6 days. His symptoms resolved but recommenced after restarting amoxicillin-clavulanate 1 week later and so his parents opted for him to receive no further antibiotics. At 4 months his lesion had improved.

Patient 5. A 7-year-old male attended the emergency department as his mother was aware of the evolving melioidosis outbreak. He had a 1 cm ulcerated, purulent skin lesion on his right arm that persisted despite 7 days of twice daily oral amoxicillin-clavulanate. A swab taken 1 week prior identified no organisms but a repeat swab isolated *B. pseudomallei*. Oral TMP-SMX was commenced but after 9 days he developed a widespread erythematous rash that improved following cessation of the drug. He was recommenced on oral amoxicillin-clavulanate three times daily, 9 days after ceasing TMP-SMX and the lesion resolved after completing a further 10 weeks of therapy.

Patient 6. A 7-year-old female attended her GP with a 1-month history of a 1cm crusted lesion on her right arm. A swab isolated *B. pseudomallei.* She was prescribed oral TMP-SMX but after 10 days developed a widespread, erythematous, pruritic rash. TMP-SMX was ceased,

her rash resolved after 4 days, and oral doxycycline was commenced. She completed a further 8 weeks of therapy and her lesion resolved.

Patient 7. A 9-year-old female attended her GP after her friend was diagnosed with cutaneous melioidosis. She had a 1 cm erythematous skin lesion over her right hip and a swab isolated *B. pseudomallei*. She was commenced on oral TMP-SMX but developed a diffuse erythematous rash 8 days later. TMP-SMX was ceased, and the rash resolved after 4 days. Oral amoxicillin-clavulanate was commenced and her lesion resolved after 12 weeks of treatment.
